# Impact of anti-doping education and doping control experience on anti-doping knowledge in Japanese university athletes: a cross-sectional study

**DOI:** 10.1186/s13011-018-0178-x

**Published:** 2018-12-05

**Authors:** Yuka Murofushi, Yujiro Kawata, Akari Kamimura, Masataka Hirosawa, Nobuto Shibata

**Affiliations:** 1 Graduate School of Health and Sports Science, Juntendo University, 1-1 Hiraka-gakuendai, Inzai-shi, Chiba, 270-1695 Japan; 2 Faculty of Health and Sports Science, Juntendo University, 1-1 Hiraka-gakuendai, Inzai-shi, Chiba, 270-1695 Japan; 3 School of Humanities, Wayo Women’s University, 2-3-1 Konodai, Ichikawa-city Chiba, 272-8533 Japan; 4 Institute of Health and Sports Science & Medicine, Juntendo University, 1-1 Hiraka-gakuendai, Inzai-shi, Chiba, 270-1695 Japan

**Keywords:** Anti-doping knowledge, University athletes, World Anti-Doping Agency’s ALPHA test

## Abstract

**Background:**

This study was conducted to elucidate the anti-doping (AD) education, doping control experience, and AD knowledge according to the World Anti-doping Code (Code) of Japanese university athletes.

**Methods:**

We collected data from 514 male athletes (M_age_ = 19.53 years, SD = 1.13) and 629 female athletes (M_age_ = 20.99 years, SD = 1.07). We asked them about their experience undergoing doping control and the AD education they had received. Then, we assessed their AD knowledge using the World Anti-Doping Agency’s Athlete Learning Program about Health and AD (ALPHA) test.

**Results:**

The results showed that 2.54% of the participants had undergone doping control. Further, 30.10% received AD education at least once, and 20.82% received AD education more than once. When comparing the ALPHA scores of athletes with/without doping test experience, we observed no significant difference. However, the ALPHA scores of athletes with/without AD education were significantly different; specifically, athletes who received AD education more than once had significantly higher ALPHA scores than non-educated athletes.

**Conclusion:**

These results revealed that doping control experience was not related to AD knowledge and that AD education was associated with AD knowledge, suggesting that athletes who receive AD education more than once have more accurate AD knowledge than less educated athletes on this topic. The importance of AD education in promoting understanding of AD according to the Code in sports is highlighted in this study.

## Background

Anti-doping (AD) education has been conducted mainly with international-level athletes up to now. Most athletes who undergo doping control are elite athletes who are participating in top national or international level competitions [1]; therefore, many athletes do not think AD applies to them. However, to realize an understanding about doping as a part of the rules of sport, AD education procedures should be implemented with all athletes. Understanding of and adherence to AD rules are essential for all athletes, regardless of their competitive level.

Henning [2] proposed an AD approach based on health promotion in amateur-level sports. Such an approach relies on providing general AD education. At present, however, the actual knowledge level of AD among all athletes is not well known. Specifically, there are no standardized investigative methods or canonicalized tests/questionnaires of AD knowledge. As Morente-Sánchez and colleagues [1] noted, the contents of existing questionnaires are insufficient when investigating AD knowledge. It seems there is a lack of available qualitative and quantitative measurements to encompass all of the AD rules similar in manner to the World Anti-doping Code (Code). Therefore, there is not much research to investigate the realities of the whole AD knowledge based on the Code.

### World anti-doping Code and responsibility as a competitor

Doping is defined as “an athlete’s use of prohibited drugs or methods to improve training and sporting results” [3]. The main activity of AD, described in the Code, is to protect the basic rights, the fairness of competition, and the health of athletes who participate in sports without doping at both international and domestic levels [4].

As of 2018, the latest AD Code is Code 2015 [4], and in this version, the role and responsibilities of athletes are emphasized more strongly than they were in earlier Codes. Among all samples analyzed by the World Anti-Doping Agency (WADA) accredited laboratories, the percentage of AD rule violations between 2015 to 2016 increased from 1.08 to 2.06% [5]. If there is an AD rule violation, strict liability is imposed regardless of whether the violation was intentional or not. On the other hand, countermeasures have been taken to provide more flexible countermeasures against unintentional violations due to inattention. When treatment is required, it is permitted to use prohibited substances after being granted approval. Here, AD rules, much like competition rules, prescribe certain conditions for competing in sports [4]. For example, in the 2016 AD testing summary, 5444 samples out of 300,565 samples were identified as being violations (1.81%). It is necessary recognized the fact that the number of athletes who are actually examined in the entire population of competitive sports is extremely few and that prevalence of doping violation among all sports participants is not 1.81%. According to the Code, athletes who are subject to doping control are primarily individuals who are participating in top national and international level competitions. However, as a prerequisite for sports participation, with or without inspection, is suggested that every athlete complies with AD rules [4]. Thus, it is critical to understand the meaning of AD precisely.

### AD education program based on the Code

As a milestone for Code 2015 fulfillment, AD research was categorized into specialized fields by Dvorak et al., including, science, medicine, education, and psychology [6]. Furthermore, priority was given to reaching a consensus on the implementation strategies of the Code 2015. Regarding the implementation of AD education at the international level, AD education has been recommended to begin from as young an age as possible as a preventive approach to achieve an environment without doping. This education should emphasize value-based education in sports. In particular, Code 2015 mentioned that national/international level athletes, who are required to undergo doping control, need to receive AD education before participating in national level competitions. Historically, AD education was administered exclusively to international-level athletes, and the content of the education focused on medical reasons for not doping, tended to be based primarily on the deterrents to doping. Since Code 2015, AD education has centered on preventive approaches under the direction of the WADA. As a preventive approach to doping, together with the Code, increasing educational awareness focused on helping athletes fully recognize sports’ intrinsic value is needed.

Further, during Code 2015 implementation, WADA developed an e-learning education system, “Athlete Learning Program about Health and AD (ALPHA)” [7] available on the Internet for athletes’ support personnel (ASP), including athletes and coaches at all competition levels. The main educational content of ALPHA is categorized into the following: 1. The doping control process; 2. Whereabouts of athletes; 3. Therapeutic use exemptions (TUE); 4. Results management; 5. Medical reasons not to dope; 6. Ethical reasons not to dope; 7. Practical help to stay clean; 8. How to deal with pressure.

ALPHA is comparatively useful as a universal AD program to understand the Code that allows for diverse AD learning among all athletes. By learning the contents of ALPHA, athletes can acquire appropriate AD knowledge and evaluate the degree of their AD knowledge based on Code. ALPHA includes 12 questions (4-Select) tests before and after e-learning to determine how much AD knowledge an athlete has in respect to the eight learning elements that cover the Code. Although previous research has included few investigations of the survey, the scope of the survey is limited to the content that is included in the Code and has been primarily limited to top international-level athletes. Additionally, there is no tool, test or questionnaire for measuring the AD knowledge that covers the entire Code. The current ALPHA test has not been sufficiently validated, but as it was prepared by researchers of WADA’s social science research program, it does suggest the presence of content validity. Thus, it has limited statistical validity, and there is sufficient scope for test reorganization in the future. Given that there is no available measure to assess knowledge of the entire Code, the ALPHA is considered to be effective to measure knowledge of AD.

In January 2018, the WADA has launched a new AD e-learning platform (ADeL) that provides access to all topics related to AD and the spirit of sport [8]. In this platform, the ALPHA is absorbed and deployed within the new system. Furthermore, ADeL offers courses for athletes, coaches, doctors, administrators, and others who are interested in learning more about AD and protecting the value of sport.

### Prior research about AD among elite athletes

Most of the research concerning AD knowledge has been conducted in samples of elite athletes who are likely to undergo doping control. Self-reported questionnaires have mainly been used to reveal the psychological characteristics of responses to doping and behavior [9, 10].

Research on AD knowledge has been reported from various viewpoints: competitive characteristics [1, 11], athletes who have violated AD rules in the past [12], cross-sectional and longitudinal investigations [13, 14], and competitors’ age [15, 16]. There have also been surveys addressing AD knowledge among ASP, such as healthcare professionals and coaches [17–20]. Most research has reported on the knowledge needed to undergo doping control, the banned substances and their adverse effects, as well as the penalties for AD rules violations. These findings reveal that athletes and ASP (except doctors) tend to have low medical knowledge regarding AD. The few existing AD studies have developed valuable surveys, but frequently the scope of the survey is not aligned with the content that is reviewed as a part of the Code. However, since 2013, some problems, such as violations of the rules due to a lack of knowledge in the context of TUE purposes, are improving because of the establishment of a system that confirms whether a banned substance is included in a prescription or commercially available medicine [21].

### AD education in Japanese educational institutions

The WADA-Code came into effect in 2004, and an enhanced system of AD has been gradually implemented since 2005 with the adoption of international conventions [22] on AD. Since 2007, the number of doping control specimens (e.g., analyzed urine and blood samples) for in and out-of-competition doping control has increased [5]. Furthermore, AD education and prevention systems have been advanced in countries since ratification of the UNESCO convention for AD, while the number of countries and sports that have become members of the Anti-Doping Agency has increased [23]. The Japanese government accepted the international convention, and has now defined AD education as a policy target in the “Sports Basic Plan” [24]. Japan, as the host country of the 2020 Tokyo Olympic and Paralympic games, has been committed to strengthening the international AD promotion system, including enhancing the international level of doping control and investigation systems, and promoting education, training, and dissemination.

As for the development of domestic AD education, in 2013, the “Olympic Movement and Doping” has been added to the subjects of health and physical education in the high school curriculum, and AD education opportunity in schools is spreading across Japan more than before [25]. Table 1 shows the content of present AD education opportunities in Japan.

**Table 1:** The content of the major anti-doping education opportunities in Japan

Types	Contents of anti-doping education	Sports					Academic	
		Ath	Co	SP	Tr	LV	Tea	Stu
Lecture Booklet	An educational implementation for athletes who are elected representatives of the country, RTPA, TPA and participate in international competitions [26].	○	○		○	I		
Booklet	Educational booklet (Play True Book) [29] of the JADA issue for the participants of the national athletic competition (prefectural opposition) including distribution and implementation of education in each province.	○	○		○	N/I		
Lecture Booklet Internet DL	Presentation of a teaching book [30] and education kit [31] on education and enlightenment from JADA for each national federation and the implementation of education for athletes at the national competition level belonging to each federation.	○	○		○	N/I		
Lecture	Anti-doping education outreach activity by JADA at national-championship-level competitions.	○	○		○	ALL		
Lecture	Conducting education that is equivalent to the curriculum guidelines for high schools. (Olympic movement and doping)	○				ALL	○	○
Lecture/Booklet	Development of certified sports pharmacist system by JADA.			○				
Booklet Internet DL	International anti-doping education package tool [32] (values and integrity of sport and anti-doping)	○				ALL	○	○
Booklet/Internet DL	Anti-Doping School Project for teachers and schools [33] (aimed at academia)	○				ALL	○	○

*Note. Ath* Athletes, *Co* Coach, *SP* Sports pharmacist, *Tr* Trainer, *Tea* Teacher, *St* Student, *LV* Level, *N* National level, *I* International level, *ALL* All levels, *DL* Download, *RTPA* Registered Testing Pool Athletes, *TPA* Testing Pool Athletes

However, the implementation of actual educational methods and the degree of individual acquisition remain unclear. Among the eight opportunities in Table 1, a nationwide common education opportunity for athletes was adopted only for the first, the RTPA (Registered Testing Pool Athletes) and TPA (Testing Pool Athletes), and the second, that is, at the national competition level [26]. In recent years, although the training materials were provided by each national sports federation and the Japan Anti-Doping Agency (JADA), the method of implementation has been entrusted to each province and has not reached a national consensus. In addition, the extent to which these educational experiences contribute to the improvement of AD knowledge has not been clarified. The guidelines for educational methods are not clearly defined because the development of AD education is ongoing.

### The position of university athletes in Japan and the need for AD knowledge

There have been few reports of AD rule violations specific to Japanese Olympic and Paralympic athletes; however, there are yearly reports on all Japanese athlete violations [4, 5]. Given the realities of Japan’s domestic sports activities, athletic clubs in secondary schools, high schools, and universities, which are affiliated with school education, appear to be the most active in sports activities, and hold competitions at various levels (e.g., district, prefectural, national, and international). Most coaches are health and physical education teachers who obtained a teacher’s license at a sports university in Japan. In other words, some Japanese university athletes will become elite athletes, coaches or physical education teachers in the future. Furthermore, among Japanese educational institutions, junior high school and high school are relatively easy to identify a route for AD education in an environment taught by teachers and exercise department leaders. However, it is more difficult to identify methods of education that are appropriate within the university environment. In this research, we focus on the level of AD knowledge in university athletes who are considering the global problems within the field of sports but may have more limited exposure to AD information.

Moreover, AD education is currently informally and arbitrarily implemented except for a small number of elite athletes. Furthermore, since the AD education implementation method is left to the site to be implemented, it is considered that the information to be received and the accumulated knowledge are different. Due to such arbitrary AD educational circumstances, there is a need to investigate the current state of AD knowledge.

### Study purpose

Given the state of the field, it is necessary to educate athletes about AD in a timely fashion, especially in Japan, and to assess the prevalence of AD education and knowledge among university athletes. The purpose of this study was to elucidate university athletes’ actual knowledge of AD as the current Code at all competitive levels and to explore the direction of AD education in the future in Japan. As such, it aimed to support the development of Japanese athletes by clarifying university athletes’ doping control experience. The results of this survey are expected to help and provide a point to educate Japanese athletes in the implementation of AD education.

## Methods

### Participants

Participants (*N* = 1143, M_age_ = 20.34 years, SD = 1.31) were 514 men (M_age_ = 19.53 years, SD = 1.13) and 629 women (M_age_ = 20.99 years, SD = 1.07), all of whom were athletes recruited across several Japanese universities. The implementation of the survey was limited to sporting events where doping-control was conducted and targeted at all athletic level athletes. Requests for surveys occurred during visits to multiple universities with sports-affiliated departments during a lecture at the school. During the visits, we conducted the ALPHA test and collected the surveys on the spot.

### Procedure

We obtained permission to conduct this study from the ethics committee of the Graduate School of Health and Sports Science, Juntendo University, Japan. The survey was conducted from September to December 2016, and the questionnaires were completed and collected during classes of each participating university. The purpose of the research was explained, and informed consent was obtained both in writing and verbally. Participants were informed that their privacy would not be compromised.

### Measures

#### Individual demographic data

Individual attributes including sex, sport, doping control experience, AD education experience (no experience, once, or more than once), duration of athletes’ career (1–5 years, 6–10 years, or ≥ 11 years), and highest level of competition for the individual and team (district, prefectural, national, or international level) were explored. Since there are no consistent methods for teaching AD, in this study, regardless of the AD education methods or format, we regarded self-reported education experience as the participants’ AD education experience.

#### Questionnaire measuring AD knowledge

To measure AD knowledge, we conducted the ALPHA test using the e-learning system, which was produced by the WADA in Japanese. The test consisted of 12 questions with four answer options (and one correct answer; Table 3, Table 6). The ALPHA score was calculated by summing correct answers (score range = 0–12). We calculated the ALPHA score and subscale score percentages and compared each to the individual attributes. After the ALPHA test, a certificate was issued if the correct answer rate was 80% or more (e.g., a score of 9.6 or more when converting to points). The correct answer rate is calculated from the number of alpha12 questions. Therefore, the evaluation in this research was measured as the achievement of a score of 9.6 or more. As a caveat, the content of the ALPHA test is attached to an e-Learning platform, so there is a limit to the available methods of data collection when compared to the measurement used in previous research. However, taking into consideration the fact that it is a test that covers the entire CODE, we carried out this survey despite these limitations to the research.

#### Analyses

We confirmed the demographic data of the sample, namely, sex, doping control experience, competition duration (years), individual competition level, team competition level, education experience, and athletic event. Next, to confirm the normality of ALPHA and subscale scores, we conducted the Kolmogorov-Smirnov test. Significant differences were observed, suggesting that the data were not normally distributed. Therefore, for subsequent analyses, we performed non-parametric tests. First, the percentage of correct ALPHA answers was calculated; then, the Mann–Whitney U test was conducted to compare ALPHA and subscale scores according to sex and doping control experience. The Kruskal-Wallis H test was conducted to compare ALPHA and subscale scores according to sports career duration, highest competition level for the individual and team, and the frequency of experiencing AD education (e.g., no experience, once, or more than once). For subsequent multiple comparisons, the Bonferroni correction was applied. In addition, we calculated effect size of each analysis and judged the magnitude of the effect size based on the criteria suggested by Cohen [27] (small = .10, medium = .30, large = .50). The significance level was set at 5%. All statistical processing was performed with SPSS Statistics 24.0 (IBM, Japan).

## Results

### Participants’ demographic data

Participants’ demographic data are shown in Table 2. The percentage of athletes who had experienced doping control was 2.54% (*n* = 29). Just under half of the athletes had no experience of AD education (49.08%; *n* = 561), while 30.10% (*n* = 344) had just one experience and 20.82% (*n* = 238) had more than once.

**Table 2:** Participants’ demographic data

	N	%
Sex		
Male	514	44.97
Female	629	55.03
Doping Control		
Experienced	29	2.54
Non-experienced	1114	97.46
Competition Duration (years)		
1–5	302	26.42
6–10	460	40.24
≥ 11	381	33.33
Individual Competition Level		
District	321	28.08
Prefectural	311	27.21
National	478	41.82
International	33	2.89
Team Competition Level		
District	259	22.66
Prefectural	227	19.86
National	595	52.06
International	62	5.42
Education Experience		
Non-educated	561	49.08
Once	344	30.10
More than once	238	20.82
Athletic Event		
Athletics	266	23.27
Football	177	15.49
Basketball	143	12.51
Baseball	93	8.14
Swimming	63	5.51
Volleyball	59	5.16
Tennis	51	4.46
Kendo	43	3.76
Softball	33	2.89
Handball	33	2.89
Badminton	22	1.92
Judo	13	1.14
Rugby	12	1.05
Gymnastics	11	0.96
Rhythmic gymnastics	10	0.87
Triathlon	9	0.79
Karate	9	0.79
Cycling	8	0.70
Futsal	6	0.52
Life guarding	6	0.52
Lacrosse	6	0.52
Ultimate frisbee	5	0.44
Ice hockey	3	0.26
American football	3	0.26
Squash	3	0.26
Table tennis	3	0.26
Naginata	2	0.17
Archery	2	0.17
Alpine skiing	1	0.09
Aerobics	1	0.09
Golf	1	0.09
Synchronized swimming	1	0.09
Skiing	1	0.09
Mountain biking	1	0.09
Competitive dance	1	0.09
Non-respondent	42	3.67
Total	1143	100

### ALPHA scores

The mean overall ALPHA score was 7.75 (± 2.30) and the correct answer rate was 64.54% (± 19.18%). The percentages of correct ALPHA answers per question are shown in Table 3.

**Table 3:** Percentage of correct ALPHA answers per question

No.	ALPHA question content		All (N = 1143)	
			% Correct	(n %)
1	What is the philosophy behind anti-doping?		74.63	85.30
	a. To restrict the pharmaceutical industry’s access to athletes	*c. To protect the spirit of sport*		
	b. To hold athletes to a higher standard than non-athletes	d. To promote discipline among athletes		
2	What is the purpose of the World Anti-Doping Code?		43.74	50.00
	a. To protect athletes’ fundamental right to participate in doping-free sports	c. To ensure harmonious and effective content anti-doping program at international level		
	b. To promote health and fairness and equality for athletes	*d. All of the above*		
3	What is the Prohibited List?		65.62	75.00
	a. The list of doctors who are not allowed to work with athletes because of doping sanctions	*c. The list of substances and methods that are prohibited in competition and out of competition*		
	b. The list of athletes that have been banned from competition	d. The list of support personnel who are not allowed to work with athletes because of doping sanctions		
4	What are the side effects of using anabolic steroids?		26.25	30.00
	a. Men with breasts and women with deep voices	c. Violent mood swings		
	b. Liver and heart failure	*d. All of the above*		
5	What does TUE stand for?		61.07	69.80
	a. Team Update Exemption	*c. Therapeutic Use Exemption*		
	b. Therapeutic Use Enhancement	d. Technical Use Exchange		
6	How can an athlete with a medical condition decide whether to take a medication?		65.18	74.50
	a. Athletes can take any medication for medicinal purposes	*c. The athlete should determine the need for the medication and seek a TUE*		
	b. The medication is permitted if the medical condition would hinder performance in competition	d. The medication is permitted if it is prescribed by a doctor		
7	Who is responsible for the substances found in an athlete’s body?		37.80	43.21
	*a. The athlete*	c. The coach		
	b. The doctor	d. The person who provided the substance		
8	What condition allows an athlete to refuse to be tested?		76.64	87.60
	a. Family commitments	c. Academic obligations		
	b. Busy schedules	*d. Athletes cannot refuse testing*		
9	When must an athlete be notified of an upcoming test?		46.81	53.50
	a. 1 month prior	c. 24 h prior		
	b. 7 days prior	*d. No advance notice is required*		
10	When do athletes have to tell their National Anti-Doping Organization where they will be living, training and competing?		37.62	43.00
	a. Athletes are not required to do this	c. During any year when the Olympics are being held		
	*b. When they are in a Registered Testing Pool (RTP)*	d. All athletes must do this		
11	What are the athlete’s right when a positive test is returned?		44.97	51.40
	a. The right to have the B sample analyzed	c. The right to copies of the laboratory documentation package		
	b. The right to attend the opening and analysis of the B sample	*d. All of the above*		
12	What is the requirement for laboratories that analyze blood or urine samples for doping control?		56.17	64.20
	a. The laboratory must be based in the country where the doping control took place	c. The laboratory must be based in the athlete’s country		
	b. Any laboratory may analyze samples	*d. The laboratory must be accredited by WADA*		

*Note*. Each correct answer is described in italic. % Correct is the correct answer rate for each question item of ALPHA. (n %) is Percentage of correct answers to the total number of athletes

A comparison of ALPHA scores revealed no significant sex differences or differences between athletes with doping control experience and those without (Table 4).

**Table 4:** Comparison of ALPHA scores by sex and doping control experience

Category	Classification	Mean	SD	median	T	U	z	*p*	η^2^
Sex	Male	7.70	2.46	8.00	293,596.0 360,200.0	161,241.00	−0.075	0.94	.00
	Female	7.77	2.16	8.00					
Doping control	Experienced	8.21	1.80	8.00	18,088.0 635,708.0	14,653.00	−0.862	0.39	.00
	Not experienced	7.73	2.31	8.00					

*Note*: ALPHA scores compared by sex and doping-control experience using Mann Whitney U test for analyzing. T is a result of signed-rank sum. η^2^ is a measure of effect size (small = .10, medium = .30, large = .50)

Table 5 shows a comparison of ALPHA scores by continuance of competition duration and individual and team competition level. A significant difference was not observed for the duration of competitive career. A significant difference was observed in individual competition level, χ^2^(3, 1139) = 9.283, *p* < .05, η^2^ = .008. Multiple comparison tests showed that scores of national-level group was lower than the district-level, *U* (1, 798) = 69,366, *p* < .05, η^2^ = .007, and international-level groups, *U* (1, 510) = 6123.5, *p* < .05, η^2^ = .009.

**Table 5:** ALPHA scores by competition duration and highest competitive level (individual and team)

Category	Classification	Mean	SD	Median	χ^2^	*df*	*p*	η^2^	Multiple comparison
Competition Duration (years)	1–5	7.89	2.32	8.00	2.038	2	0.361	0.002	*n.s*
	6–10	7.74	2.30	8.00					
	≥11	7.64	2.28	8.00					
Individual Competition Level	District	7.93	2.27	8.00	9.283	3	0.026	0.008	District > National (*p* = 0.020, η^2^ = 0.007) International > National (*p* = 0.030, η^2^ = 0.009)
	Prefectural	7.83	2.31	8.00					
	National	7.52	2.31	8.00					
	International	8.39	2.21	9.00					
Affiliated Team Competion Level	District	7.90	2.31	8.00	7.795	3	0.050	0.006	*n.s.*
	Prefectural	7.90	2.25	8.00					
	National	7.58	2.31	8.00					
	International	8.08	2.36	9.00					

*Note*. ALPHA scores compared between competition duration, individual competition level, and affiliated team competition level using Kruskal-Wallis test for analyzing, and subsequent multiple comparisons were calculated using Mann Whitney U test. η^2^ is effect size (small = .10, medium = .30, large = .50)

### ALPHA scores compared with AD education frequency

**Fig. 1:**
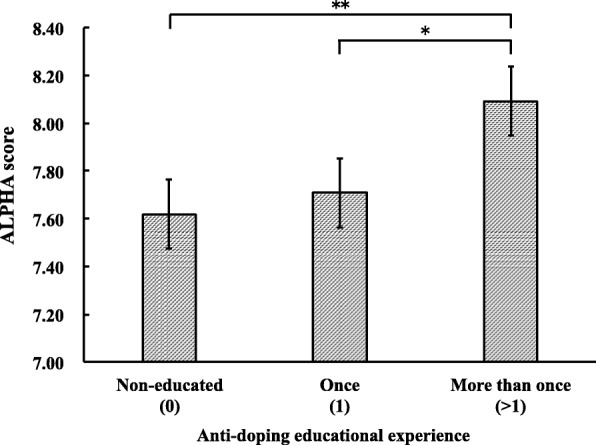
Comparison of anti-doping education experiences and ALPHA scores. *p < .05, ** *p* < .01

We compared ALPHA score by participants’ experience of AD education across three groups: non-educated, educated once, and educated more than once (Figure 1). Results showed that a significant difference was observed between groups, χ^2^(2, 1143) = 8.097, *p* < .05. Subsequent multiple comparisons demonstrated that the participants who were educated more than once (M = 8.09, 67.43%, SD = 2.36, 19.68%) had significantly higher ALPHA scores than the non-educated group (M = 7.62, 63.49%, SD = 2.36, 19.80%), *U* (1, 798) = 58,837, *p* < .01, η^2^ = .009, and the educated once group (M = 7.71, 64.24%, SD = 2.11, 17.61%) *U* (1, 581) = 36,089, *p* < .05, η^2^ = .01.

The correct answer rate for each of the 12 ALPHA test questions according to the frequency of AD education experience is shown in Table 6.

**Table 6:** Percentage of correct ALPHA answers according to educational frequency

	ALPHA question content		Education (frequency)					
No			Non-educated (n = 561)		Once (n = 344)		More than once (n = 238)	
			Correct %	(n %)	Correct %	(n %)	Correct %	(n %)
1	What is the philosophy behind anti-doping?		72.97	83.40	74.54	85.20	78.65	89.90
	a. To restrict the pharmaceutical industry’s access to athletes	*c. To protect the spirit of sport*						
	b. To hold athletes to a higher standard than non-athletes	d. To promote discipline among athletes						
2	What is the purpose of the World Anti-Doping Code?		40.51	46.30	46.28	52.90	47.77	54.60
	a. To protect athletes’ fundamental right to participate in doping-free sports	c. To ensure harmonious and effective content anti-doping program at international level						
	b. To promote health and fairness and equality for athletes	*d. All of the above*						
3	What is the Prohibited List?		63.60	72.70	67.37	77.00	67.63	77.30
	a. The list of doctors who are not allowed to work with athletes because of doping sanctions	*c. The list of substances and methods that are prohibited in competition and out of competition*						
	b. The list of athletes that have been banned from competition	d. The list of support personnel who are not allowed to work with athletes because of doping sanctions						
4	What are the side effects of using anabolic steroids?		25.90	29.60	24.41	27.90	29.75	34.00
	a. Men with breasts and women with deep voices	c. Violent mood swings						
	b. Liver and heart failure	*d. All of the above*						
5	What does TUE stand for?		61.07	68.30	60.80	69.50	64.65	73.90
	a. Team Update Exemption	*c. Therapeutic Use Exemption*						
	b. Therapeutic Use Enhancement	d. Technical Use Exchange						
6	How can an athlete with a medical condition decide whether to take a medication?		65.62	75.00	62.82	71.80	67.28	76.90
	a. Athletes can take any medication for medicinal purposes	*c. The athlete should determine the need for the medication and seek a TUE*						
	b. The medication is permitted if the medical condition would hinder performance in competition	d. The medication is permitted if it is prescribed by a doctor						
7	Who is responsible for the substances found in an athlete’s body?		35.87	41.00	28.96	33.10	32.37	37.00
	*a. The athlete*	c. The coach						
	b. The doctor	d. The person who provided the substance						
8	What condition allows an athlete to refuse to be tested?		76.90	87.90	74.80	85.50	78.65	89.90
	a. Family commitments	c. Academic obligations						
	b. Busy schedules	*d. Athletes cannot refuse testing*						
9	When must an athlete be notified of an upcoming test?		45.06	51.50	46.81	53.50	50.74	58.00
	a. 1 month prior	c. 24 h prior						
	b. 7 days prior	*d. No advance notice is required*						
10	When do athletes have to tell their National Anti-Doping Organization where they will be living, training and competing?		39.02	44.60	36.92	42.20	35.70	40.80
	a. Athletes are not required to do this	c. During any year when the Olympics are being held						
	*b. When they are in a Registered Testing Pool (RTP)*	d. All athletes must do this						
11	What are the athlete’s right when a positive test is returned?		46.02	52.60	42.26	48.30	46.28	52.90
	a. The right to have the B sample analyzed	c. The right to copies of the laboratory documentation package						
	b. The right to attend the opening and analysis of the B sample	*d. All of the above*						
12	What is the requirement for laboratories that analyze blood or urine samples for doping control?		54.24	62.00	58.01	66.30	58.09	66.40
	a. The laboratory must be based in the country where the doping control took place	c. The laboratory must be based in the athlete’s country						
	b. Any laboratory may analyze samples	*d. The laboratory must be accredited by WADA*						

*Note*. Each correct answer is described in italic. % Correct is the correct answer rate for each question item of ALPHA. (n %) is Percentage of correct answers to the total number of athletes

## Discussion

### AD knowledge among Japanese university athletes

Overall, university athletes had a low rate of correct answers on the ALPHA test, especially considering that all athletes must be familiar with the principles of Code 2015 and AD basic principles during athletic competitions. Regarding specific items, the high correct answer rates for items 1 and 8 indicate that university athletes understand that AD is necessary to protect the spirit of the sport and that there is a need to cooperate in doping control. However, such questions include aspects of information that have a possibility of having social desirability responses, and these factors should be considered when conducting AD education [28]. On the other hand, athletes’ lack of knowledge on the medical reasons for not doping has been identified among elite athletes [1], and Japanese university athletes appear to have a similar level of awareness (cf. item 4: side effect). In addition, the athletes’ responsibility (cf. item7: responsible when the substances found in an athlete’s body) based on the Code 2015 was not well understood by the athletes in the sample.

### The relationship between athletes’ demographics and ALPHA scores

Results showed that there were no significant differences in ALPHA scores by sex or doping control experience. As for the absence of significant gender differences in knowledge, it can be understood that AD education may be equally implemented regardless of student gender. Furthermore, in the implementation and survey of future AD education, it would be preferable to ensure the equal opportunities for each gender with regard to the survey results. In this study, the number of athletes who had doping control experience was very small compared to those with no experience. Regarding the fact that there was no significant difference in the presence or absence of doping control experience, doping control experience itself may not contribute to the acquisition of AD knowledge. Therefore, educational implementation and surveys of international level athletes are necessary. It is also necessary to investigate AD knowledge level among athletes with doping control experience in future research. In the analysis of differences between the team competition level, no significant difference was shown at any competition level. Analysis data revealed that even if the competition level of their team was high, it would not lead to high knowledge of individual AD. In future AD education and research, there is a need to focus on each athlete’s AD knowledge, not on their level of competition.

On the other hand, individual competition level did have a significant impact on ALPHA scores, revealing that the district- and international-level groups had significantly higher scores than the national-level group. The reasons for this finding should be clarified in future research. The poor knowledge of athletes competing in the national-level group might indicate a serious problem. This result may be due to several reasons. For example, as seen in RTPA and TPA in Table 1, the International-level group seems to be the most knowledgeable in AD. National-level athletes also receive greater support compared to athletes at lower competition level from management system such as ASP. National-level athletes may not have a strong sense of ownership compared with international level athletes because they have less of a chance of taking doping control. The effect size for the difference between national level and international level athletes was small, but this difference cannot be ignored. Although the goal of enhancing competitiveness at all competition levels remains unchanged, national-level athletes are considered to be more devoted and focused to their competitive sports activities than are the district-level athletes. For that reason, national-level athletes may pay considerable attention only daily training, rather than being interested in their required responsibilities and roles. This point should be clarified in future research.

### The relationship between ALPHA scores and the frequency of AD education among athletes

Notably, the frequency of AD education positively affected AD knowledge. University athletes who were educated once and more than once had higher levels of AD knowledge compared to those who were non-educated. This suggests that there is a need to implement AD education more than once with university athletes to improve their AD knowledge. However, as a point of caution, overall ALPHA scores were still relatively low, compared to the desired evaluation score of 9.6 or more even among those athletes who were educated more than once. At this time, the course contents of AD education have not been verified, and it is difficult to confirm whether AD education at the individual level is appropriate.

### Towards improving AD education in Japanese universities

This study elucidated the reasons that AD knowledge is critical for Japanese university athletes and those who wish to become coaches in the future. Furthermore, it clarifies the direction for future AD education. In Japan, AD education is gradually being implemented. However, AD education including ALPHA is often optional for athletes who are not competing at the highest level, and the results for university athletes demonstrate this pattern. Furthermore, voluntary education on AD may have challenges with covering all aspects of the Code, and the university athletes have pointed out that it is difficult to possess knowledge of all of the AD rules. Specifically, the following three points were illustrated: 1) Japanese university athletes have tended to have insufficient AD knowledge; 2) athletes who have experience competing at the national competition level have the possibility of lying a problem as the AD rule violations such as having insufficient AD knowledge compared with other competition level athletes; and 3) to acquire sufficient AD knowledge, athletes may gain and retain greater levels of AD knowledge by receiving AD education more than once. More consideration for a second point, athletes who are competing in national level competition, which are realistically likely to undergo doping-control, are expected to have sufficient knowledge of AD rules in order to fulfill their responsibilities as an athlete identified in the Code and on ALPHA test item 7. Moreover, based upon the results of lowest scoring ALPHA test item, education about medical issues (cf. item 4: side effect), the latest information about AD, and athletes’ responsibilities (cf. item 7: responsible when the substances found in an athlete’s body) is recommended to be strengthened among university athletes. These points would be the future direction of AD education implementation.

Based upon the results of this study, we suggest the need to construct a system to experience the AD education more than once at each university where Japanese university athlete is enrolled. As the prohibited list is updated annually on January 1, and there are other revisions and changes occur yearly, it is necessary to obtain the latest rules and information and to consider the implementation timing of the AD education.

## Conclusions

In this study, the usefulness of AD education and the need for educational interventions were found. AD education based on the Code 2015 should be implemented for all athletes, regardless of competitive level, and internationally/nationally competition level athletes appeared to be particularly educated about doping control. During AD education, the development of teaching material should consider the latest medical knowledge related to AD. Furthermore, in line with the current Code, it is necessary to create and standardize world-class learning content that can enhance individuals’ knowledge of AD. In the future, it will be necessary to examine the effects of WADA-ALPHA e-learning and the latest WADA educational content to consider proposals to address more specific issues in AD education. On the other hand, the effectiveness of education is limited. In order to promote AD activities as shown in Dvorak et al. [6], items indicated by the key components of the implementation of the Code 2015 and ALPHA, it should be noted that there is a need to implement AD education in combination with other methods of reducing doping beyond general awareness by athletes. To protect the intrinsic value and integrity of sports, we will further explore how to intervene in AD education, which will hopefully enhance international AD education.

## Abbreviations

AD: Anti-doping
ADeL: Anti-doping e-learning platform
ALPHA: Athlete Learning Program about Health and AD
ASP: Athletes’ support personnel
Code: World anti-doping code
JADA: Japan anti-doping agency
UNESCO: The United Nations Educational, Scientific and Cultural Organization
WADA: World anti-doping agency
